# Estimating and projecting the number of new HIV diagnoses and incidence in Spectrum's case surveillance and vital registration tool

**DOI:** 10.1097/QAD.0000000000002324

**Published:** 2019-08-02

**Authors:** Severin G. Mahiane, Kimberly Marsh, Robert Glaubius, Jeffrey W. Eaton

**Affiliations:** a Center for Modeling and Analysis, Avenir Health, Glastonbury, Connecticut, USA; b Strategic Information Department, UNAIDS, Geneva, Switzerland; c Department of Infectious Disease Epidemiology, Imperial College London, London, UK.

**Keywords:** AIDS-related mortality, HIV case-based surveillance., HIV incidence, statistical models

## Abstract

**Objective:**

The Joint United Nations Programme on HIV/AIDS-supported Spectrum software package is used by most countries worldwide to monitor the HIV epidemic. In Spectrum, HIV incidence trends among adults (aged 15–49 years) are derived by either fitting to seroprevalence surveillance and survey data or generating curves consistent with case surveillance and vital registration data, such as historical trends in the number of newly diagnosed infections or AIDS-related deaths. This article describes development and application of the case surveillance and vital registration (CSAVR) tool for the 2019 estimate round.

**Methods:**

Incidence in CSAVR is either estimated directly using single logistic, double logistic, or spline functions, or indirectly via the ‘r-logistic’ model, which represents the (log-transformed) per-capita transmission rate using a logistic function. The propensity to get diagnosed is assumed to be monotonic, following a Gamma cumulative distribution function and proportional to mortality as a function of time since infection. Model parameters are estimated from a combination of historical surveillance data on newly reported HIV cases, mean CD4^+^ at HIV diagnosis and estimates of AIDS-related deaths from vital registration systems. Bayesian calibration is used to identify the best fitting incidence trend and uncertainty bounds.

**Results:**

We used CSAVR to estimate HIV incidence, number of new diagnoses, mean CD4^+^ at diagnosis and the proportion undiagnosed in 31 European, Latin American, Middle Eastern, and Asian-Pacific countries. The spline model appeared to provide the best fit in most countries (45%), followed by the r-logistic (25%), double logistic (25%), and single logistic models. The proportion of HIV-positive people who knew their status increased from about 0.31 [interquartile range (IQR): 0.10–0.45] in 1990 to about 0.77 (IQR: 0.50–0.89) in 2017. The mean CD4^+^ at diagnosis appeared to be stable, at around 410 cells/μl (IQR: 224–567) in 1990 and 373 cells/μl (IQR: 174–475) by 2017.

**Conclusion:**

Robust case surveillance and vital registration data are routinely available in many middle-income and high-income countries while HIV seroprevalence surveillance and survey data may be scarce. In these countries, CSAVR offers a simpler, improved approach to estimating and projecting trends in both HIV incidence and knowledge of HIV status.

## Abstract 2

Supplemental Digital Content is available in the text

## Introduction

The Joint United Nations Programme on HIV/AIDS (UNAIDS) works with country partners to produce global, regional, and country-specific estimates of HIV burden annually to guide national and global planning and monitoring [1,2]. Methods, tools, and assumptions that underpin these estimates are developed with guidance from the UNAIDS Reference Group on Estimates, Modelling and Projections, which works to advance the development of statistical and mathematical approaches to modelling the HIV epidemic [3]. Most countries use Spectrum, a UNAIDS-supported modelling software tool, to generate these annual estimates.

The case surveillance and vital registration (CSAVR) tool, first introduced in Spectrum in 2014 under the name fit to program data, was developed as an alternative curve fitting tool to the Estimates and Projection Package (EPP) for countries with robust historical vital registration and case-based HIV surveillance systems. The tool was used in 2019 to estimate HIV incidence trends among adults aged 15–49 years in 43 of the 170 countries that contribute to UNAIDS regional and global estimates, an increase from 2014 where it was used by just 16 countries. Before 2014, most country models relied on EPP to derive national incidence curves from HIV seroprevalence surveillance and survey data among key populations at higher risk of HIV exposure (such as female sex workers, gay men, and other MSM and people who inject drugs) and pregnant women attending antenatal care clinics, alongside estimates of the size of each population subgroup [1,4–6], supplemental file.) EPP-derived estimates have been critiqued in settings where prevalence data among key populations are not routinely available, not nationally representative, or where accurate key population size estimates are not available [7,8]. In countries with very low-level epidemics where few historical, repeated measures of HIV prevalence are available among key populations, estimation of HIV incidence using EPP was not possible.

The CSAVR tool overcomes the challenges of scarce HIV serosurveillance and survey data and key population size estimates by offering countries with robust vital registration and case-based HIV surveillance systems an alternative approach for deriving HIV incidence estimates from these data. Many middle-income and high-income countries have reasonably complete vital registration systems [9] and low misclassification of AIDS-related deaths to other causes [9,10]. Although published evaluations of the quality and completeness of HIV case surveillance systems are more limited, studies have shown that HIV incidence curves derived from robust case surveillance data are a good alternative to fits in EPP [11,12].

A key challenge of CSAVR and other back-calculation approaches to estimating incidence is that the number of new diagnoses is assumed to represent past incidence. Therefore, either estimates of time from infection to diagnosis or the proportion of HIV-positive people who die undiagnosed are needed to accurately infer past incidence from new diagnoses. In previous iterations of the CSAVR tool, users were required to enter information about the expected time from infection to diagnosis and the proportion of HIV-positive people who died undiagnosed; however, in practice, that information was typically not available. However, data that are usually available in many countries are CD4^+^ cell count at diagnosis. This measure has been shown to be a reasonably good proxy for time since infection at the population level [1].

In the 2017 version of CSAVR, we improved the tool by developing an approach to fitting incidence that uses Spectrum's AIDS Impact Model key assumptions and available information on number of new diagnoses, deaths and/or mean CD4^+^ at diagnosis to estimate incidence trends, mean time from infection to diagnosis, CD4^+^ at diagnosis and the proportion of people living with HIV who have been diagnosed over time. This article describes this extension as well as other advances in the development of methods and a new approach for incorporating uncertainty in 2019 estimates.

## Methods

### Modelling HIV incidence among adults aged 15–49 years

Previous versions of CSAVR used the double logistic or single logistic family of parametric functions to model HIV incidence over the course of the epidemic [1]. This family was suitably flexible to capture HIV epidemic trends in many countries. However, this family was inadequate for several countries, for example, countries where data suggested more than one wave of infection. To address this limitation, two additional options were made available in Spectrum for 2019: a semiparametric spline and a single logistic function for the HIV transmission rate, termed ‘r-logistic’.

#### Double logistic curve

In CSAVR, HIV incidence may be modelled as a double logistic function: 
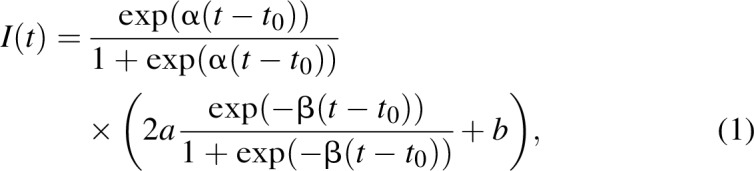


where α > 0 defines the initial epidemic growth, *a* > 0 modulates the peak incidence level, *b* > 0 defines the asymptote (i.e., the incidence value over time), β > 0 defines the rate of convergence to the asymptote, and *t*_0_ > 0 is a location parameter, at which the value of the function is (*a* + *b*)/2. Many infectious disease models either lead to the double logistic function or to functions that closely resemble it. We fitted this model with the following prior distribution on its parameters: 
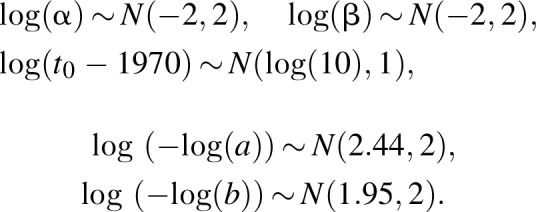


The double logistic curve [Eq. (1)] was first proposed by Stover *et al.*[11] and describes a flexible class of functions. However, these functions assume that incidence eventually converges to a constant level, which is not consistent with case notification data in some countries. Alternately, in settings with very scarce case surveillance and vital registration data, the parameter dimension may be too large for incidence to be well identified.

#### Single logistic curve

When there is no evidence of an inflection point in case notifications or there are too few data points, a single logistic curve may be warranted. In the 2016 version of CSAVR, we added a second option that models incidence as follows: 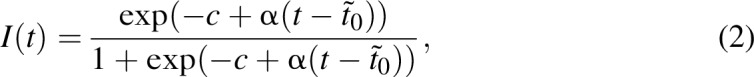


where 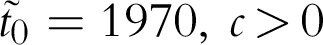, *c* > 0 defines the incidence at time 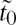 and α > 0 defines the rate of increase of the trend. We fitted this model, which is a straightforward extension of cumulative form of logistic function, with the following prior distributions on its parameters: 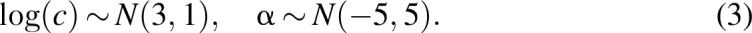


#### Splines

We included second order segmented polynomial functions as a more flexible alternative to the single and double logistic functions. That class of functions was first proposed for age-specific HIV incidence estimation by Mahiane *et al.*[13] and belongs to the wide family of splines. In the approach considered here, we set the number of knots to three and estimate their positions. Although this family of functions is very flexible, the number of parameters needed can be relatively large and are not naturally constrained to be nonnegative. To overcome these limitations, we transformed the spline, modelling incidence as follows: 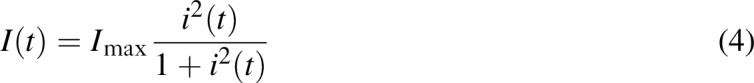


where *I*_max_ is the largest possible value allowed for the incidence rate, 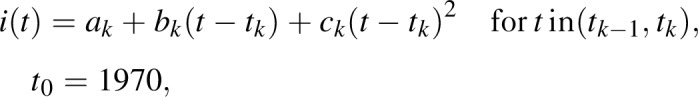


and *a*_*k*_,*b*_*k*_,*k* = 0…3 and *t*_*k*_,*k* = 1…3 are parameters to be estimated. The model is fitted with the following prior distribution on its parameters: 
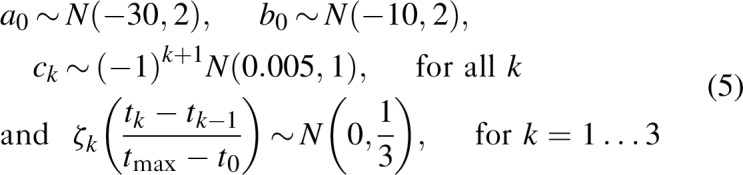


where *t*_max_ is the final year of the projection and *ζ* = (*ζ*_1_,*ζ*_2_,*ζ*_2_) is the inverse of the transformation 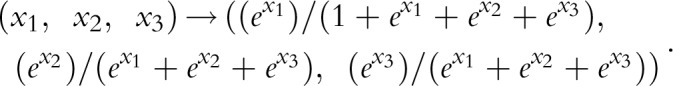.

#### Transmission model using the ‘r-logistic’ function

Instead of directly modelling the HIV incidence rate, we can also model the transmission rate *r*(*t*) as in the EPP model [14]. In this case, the incidence rate is given by 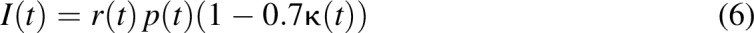


where *p*(*t*) is the prevalence at time *t*, κ is the antiretroviral therapy (ART) coverage, and 0.7 is the average reduction in transmission per additional person on ART. We use a logistic function to model the logarithm of *r*(*t*), termed *rlogistic* with four parameters: 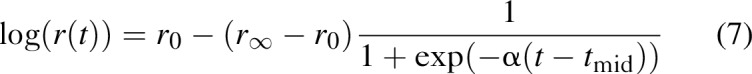


where exp (*r*_0_) is the initial exponential growth rate of the epidemic, exp (*r*_*∞*_) is the equilibrium value for *r*(*t*), *α* is the rate of change of *r*(*t*) in the log-scale and *t*_mid_ is the inflection point. For this model, we specify a fifth parameter, *ι*, as the incidence rate at time *t* = *t*_0_, providing the initial pulse of infections. This model is fitted with the following prior distributions on its parameters: 
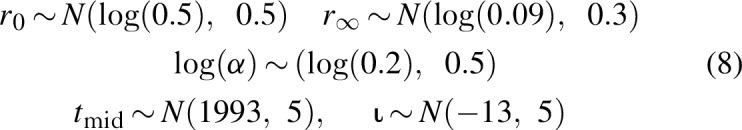


### Modelling the diagnosis rate, mean CD4^+^ cell count at infection and mean time from infection to diagnosis

We assume that the diagnosis rate is proportional to mortality rate in absence of treatment (i.e., the rate at which individuals aged *a*, infected at time *w* are diagnosed at time *v*) increases over time and that the rate of diagnosis by CD4^+^ category increases proportional to mortality rate in absence of treatment, which is given by the formula:


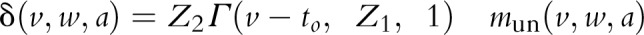
 where Γ is the Gamma cumulative distribution function with shape *z*_1_, *z*_2_ is a scale factor, and *t*_0_ is the first year of diagnosis, and *m*_un_ is the mortality rate as a function of time and age at infection.

We obtain the CD4^+^ trajectory as a function of age at infection and duration of infection using Spectrum's assumptions on CD4^+^ progressions rates and CD4^+^ distribution at infection. Details of the approach can be found in the Supporting Information. Finally, we assume that, when ART becomes available, individuals who are diagnosed initiate treatment at a rate 3 exp(*∊*)/1 + exp(*∊*), before they reach WHO eligibility criteria.

We assigned relatively weak priors to the diagnostic parameters as described below: 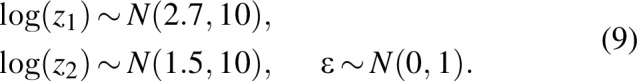


### Estimation procedures

The model estimates the following parameters *θ* = (*θ*′,*z*_1_,*z*_2_,*∊*), where *θ*′is the component related to the selected functional form for incidence (i.e., the parameters determining the shape of the single or double logistic, segmented polynomials, or r-logistic models) and *z*_1_,*z*_2_,*∊* are the parameters determining diagnosis and treatment initiation.

Let us assume that data consist of numbers of new diagnoses 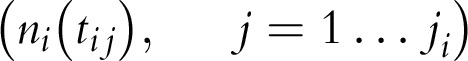, deaths 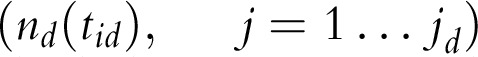, and number of people on ART 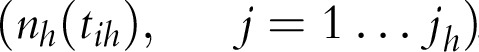, where 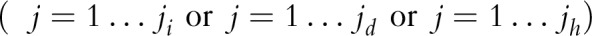 are observation times and follow Poisson distributions. We further assume that the observed CD4^+^ cell counts at diagnosis follow a Gamma distribution and that the mean CD4^+^ cell count at diagnosis can only be measured in years when there is at least one new diagnosis. Then, if *g*_*k*_(*t*_*kj*_), *j* = 1…*j*_*k*_ is the mean CD4^+^ cell count observed at times *j* = 1…*j*_*k*_, the loss function is given by 
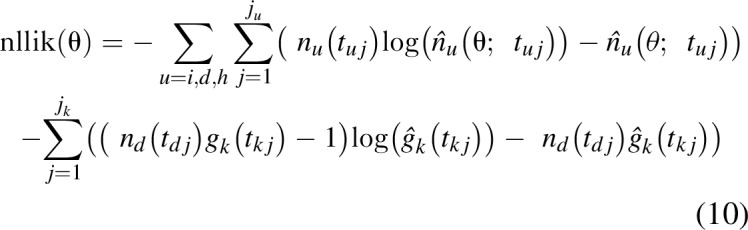


We adjusted the parameters by maximizing the posterior distribution, which is equivalent to minimizing 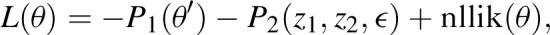


where *P*_1_ is the log prior distribution for the incidence model parameters *θ*′ determined by [Eq. (3), Eq. (5) or Eq. (7)], and *P*_2_ is the log prior distribution for the diagnosis model parameters determined by [Eq. (9)].

We used the Kernel Hamiltonian Monte Carlo [15] approach for a full Bayesian calibration. Our preliminary analyses suggested that 2000 burn-in samples were necessary. We stopped the procedure when the number of accepted samples reached 1000.

Akaike information criterion (AIC) [16] was used for model selection. For each candidate model, AIC (θ) = 2 nllik (θ)+2 *p* where *p* is the dimension of the parameter, was evaluated at the parameter minimizing Eq. (10) then, the model with the smallest AIC was chosen; that is, estimates obtained using this latter model were used for estimations and projections.

### Analysis

We applied the new CSAVR model to data from 31 countries (Table 1) which used CSAVR during the 2018 UNAIDS estimates round. Countries were selected for inclusion on the basis that they have high-quality vital registration since 1980, which is a robust source of data for HIV deaths [9]. Raw AIDS-related deaths among all ages from the vital registration system in the 2018 Spectrum files were replaced with the most recent estimates of AIDS-related deaths adjusted for incompleteness and misclassification among adults 15 years and older from the Institute for Heath Metrics and Evaluation Global Burden of Disease study or the WHO. Preference was given to IMHE estimates, which provided a longer time series of data from 1990 compared with WHO where published estimates are available only from 2000. Modelled estimates rather than raw numbers of AIDS-related deaths were used in CSAVR starting in 2019.

**Table 1:** Best incidence model^a^ for countries included in the analysis.

Eastern Europe	Western Europe	Latin America and the Caribbean	Other
Czech Republic^3^	Austria^3^	Costa Rica^4^	Australia^1^
Estonia^1^	Greece^4^	Panama^4^	Israel^1^
Hungary^3^	Finland^1^	Cuba^2^	Japan^3^
Latvia^4^	Iceland^3^	Bahamas^3^	Kuwait^3^
Poland^4^	Ireland^1^	Belize^1^	New Zealand^4^
Romania^3^	Sweden^3^	Argentina^1^	
	Spain^3^	Barbados^4^	
	Luxembourg^2^	Chile^3^	
	Switzerland^4^	Mexico^3^	
	Portugal^1^		
	Norway^3^		

^1^Double logistic curve; ^2^logistic curve; ^3^spline; ^4^r-logistic.

^a^Best incidence model chosen based on the AIC. AIC, Akaike information criterion.

## Results

**Fig. 1:**
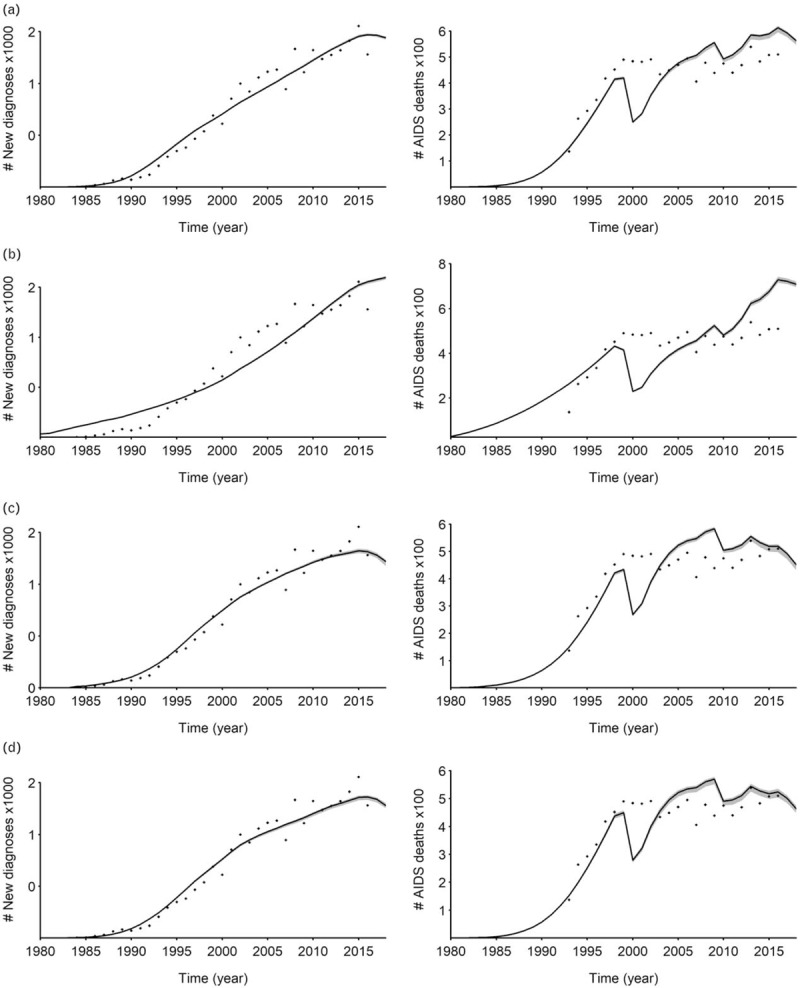
Numbers of new HIV diagnoses and AIDS deaths in Panama.

We fitted the four incidence models (double logistic, single logistic, segmented polynomial, and r-logistic) for each country. The fits are illustrated with data from Panama in Fig. 1. Table 1 shows the best model for each country included in the analysis. Based on the AIC, the spline model for incidence appeared to provide the best fit in most countries (45%), followed by the r-logistic (25%), the double logistic (25%), and the single logistic models.

Dots show the reported numbers of (left column) new diagnoses or (right column) AIDS deaths. Solid lines show model fits using (a) double logistic, (b) single logistic, (c) spline, or (d) r-logistic curves. Grey regions show the 95% credible regions around model fits.

**Fig. 2:**
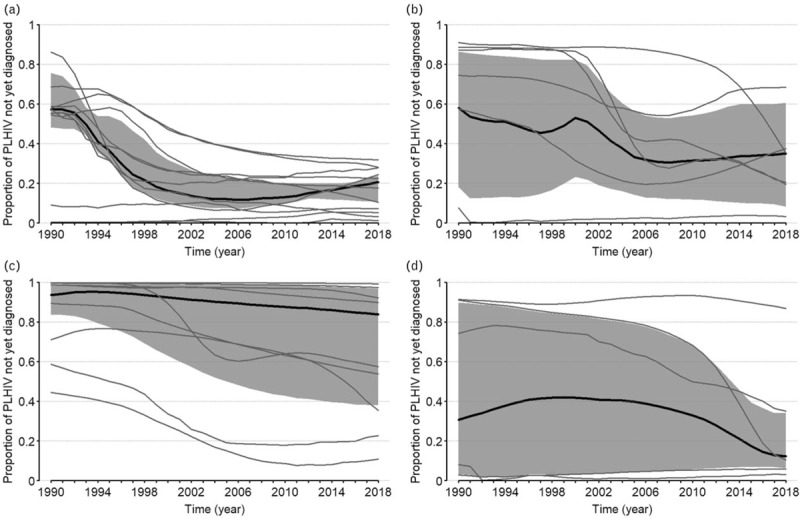
Trends of the distributions of the proportion of people living with HIV who have not been diagnosed yet by regions (a) Western Europe, (b) Eastern Europe, (c) Latin America and Caribbean, (d) Others.

The proportion of HIV-positive people who knew their status increased from about 0.31 [interquartile range (IQR): 0.10–0.45] in 1990 to about 0.77 (IQR: 0.50–0.89) in 2017. Figure 2a–d display the trends of the distributions of the proportions of people living with HIV who do not know their statuses as a function of time, for Western European, Eastern European, Latin America and Caribbean, and other countries, respectively, together with the regions’ aggregated estimates and their 95% confidence regions.

The grey lines represent countries trends, the solid black lines represent the regions’ aggregated medians and their 95 confidence regions obtained using the bootstrap resampling method represented by the greyed areas.

They suggest a decrease of the proportion of HIV-infected and undiagnosed individuals in most countries in Western Europe, with the aggregated proportion decreasing from about 43% [95% confidence interval (CI): 25–52%] in 1990 to 80% (95% CI: 78–89%) in 2017. Aggregated proportions are very noisy in the other regions. However, the sample of countries (9) in Latin America and the Caribbean region appeared to have the largest cohort of countries with less than 60% of HIV-infected people knowing their status.

**Fig. 3:**
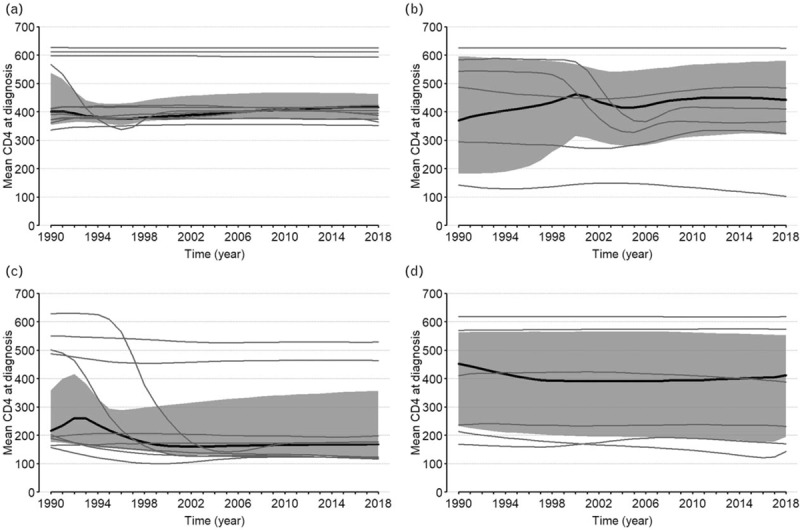
Trends of mean CD4^+^ at diagnosis by regions (a) Western Europe, (b) Eastern Europe, (c) Latin America and Caribbean, (d) Others.

The mean CD4^+^ at diagnosis across countries appeared to be stable, decreasing from 410 cells/μl (IQR: 224–567) in 1990 to 373 cells/μl (IQR: 174–475). Figure 3a–d display the trends of the distributions of the mean CD4^+^ cell count at diagnoses for Western European, Eastern European, Latin America and Caribbean, and other countries, respectively, together with the regions’ aggregated estimates and their 95% confidence regions. They suggest that the estimated mean CD4^+^ at diagnosis has been stable since 1990 and that levels are similar across regions, except in Latin America and Caribbean.

The grey lines represent countries trends, the solid black lines represent the regions’ aggregated medians and their 95 confidence regions obtained using the bootstrap resampling method represented by the greyed areas.

## Discussion

In this article, we reviewed the CSAVR methods and described the recent model developments for Spectrum in 2019. The newly added r-logistic model and spline for incidence improved fits to the program data in eleven countries. Overall, our results suggested an increase of status awareness and a decrease in mean CD4^+^ at diagnosis. This may imply that most of the newly diagnosed individuals have been infected for a longer period. The aggregated estimates presented in this study do not represent regional estimates because the analysis was restricted to countries with medium-to-high-quality vital registration data.

The use of CSAVR, with its ongoing expansion and improvements in methods, has offered a number of benefits. Perhaps the most important for countries is the transparent fitting process to routinely available surveillance and vital registration data and the acceptability of modelled results. Another benefit of CSAVR as implemented in 2019 compared with 2016 is that some assumptions were relaxed. For example, information on the estimated time from infection to diagnosis or the proportion of HIV-positive individuals who died undiagnosed is no longer required from the user as an input but is instead estimated in the fitting process. Uncertainty was also previously obtained using asymptotic properties of the maximum likelihood estimation method, which failed under some conditions, whereas in this round, the Bayesian calibration offered a more robust approach by incorporating prior information.

The tool has some limitations and additional work is planned to improve the CSAVR tool and its application by countries in future estimation rounds. One of the main priorities will be to work with countries to document the quality and completeness of program data inputs and to more accurately incorporate uncertainty in the inputs into the uncertainty bounds around the final incidence estimates. It might also be useful document the events associated with sequentially dated events and how these event-times are ascertained (e.g., prospectively or retrospectively). These are part of the data generating process that also need to be statistically modelled. However, this will demand more resources and due diligence.

The spline curve appeared to fit data the best in more countries than any other options available in the tool. It has the advantage of producing flexible curves to fit to time-series data that are not confined to specific functional shapes as other parametric models. However, although the AIC was chosen for model selection, this option is not immune to overfitting. In fact, the spline model implemented (with three knots) uses more parameters than its concurrent model. The possibility for users to try different number of knots or to use generalizations of logistic and double logistic options will be explored in future work.

The current version of the tool only uses information from adult populations. Furthermore, the tool assumes that migration does not differ by HIV status. This may not be the case in some settings, especially when there is a large influx of refugees from countries with higher or lower HIV prevalence. Recent studies in Colombia, Australia, and some European countries, reported for example, higher HIV prevalence among migrants [17–19]. Because the models used belong to the family of back-calculation methods, assigning a wrong place of infection to migrants can lead to wrong past incidence which in turn leads to wrong estimates and projection of AIDS deaths.

The models also assumed that the propensity to be tested increased with AIDS-related mortality and that the diagnosis rate over time increases and stabilizes proportional to a simple parametric form given by the cumulative gamma function. Although this assumption seems reasonable, it is possible that testing campaigns makes the model unsuitable for some years. Nevertheless, our estimates seem to agree with those obtained from other models. For instance, our analysis suggested that about 17% of infected individuals in Western Europe didn’t know their status in 2016; this is comparable with the 15% estimated for the same year Centre for Disease Prevention and control/van Sighem *et al.*[20], for the European Union and European Economic Area. Improvements are needed to account for information on HIV-related migrations, new diagnoses among children, and/or testing campaigns, when available. These developments are left for future research.

Another area for future work is to explore how countries might produce HIV incidence curves by key populations or within smaller level geographic areas using this tool. Although the functionality could be incorporated within the model, it would require countries to introduce changes in case notification forms that capture the likely location and suspected route of transmission of the infection. As countries begin to realize the benefits of using case reporting and vital registration system data to produce more robust estimates of the impact of the HIV epidemic, the level of effort required to achieve more granular estimates may not seem so extraordinary.

## Acknowledgements

The authors wish to thank UNAIDS for ongoing support for development of Spectrum. JWE acknowledges funding support from UNAIDS, NIH R01-AI136664-01, and joint Centre funding from the UK Medical Research Council and Department for International Development, MR/R015600/1.

### Authors contributions

Conceived, designed, and performed the experiments: S.G.M., R.G., K.M., J.W.E. Analysed the data: S.G.M., K.M., R.G., J.W.E. Wrote the first draft of the article: S.G.M., R.G., K.M. Contributed to the writing of the article: S.G.M., J.W.E., R.G., K.M. Agree with the article's result and conclusion: S.G.M., J.W.E., R.G., K.M.

### Conflicts of interest

There are no conflicts of interest.

## Supplementary Material

Supplemental Digital Content
