# Supplementary material for: Estimating and projecting the number of new HIV diagnoses and incidence in Spectrum's case surveillance and vital registration tool
Source: AIDS. 2019 Aug 2;33(Suppl 3):S245–53. doi: 10.1097/QAD.0000000000002324 (PMC6919234; doi:10.1097/QAD.0000000000002324)
Supplement: Supplemental Digital Content [file aids-33-s245-s001.pdf]

Supporting information for: Estimating and Projecting the number of new HIV diagnoses and Incidence in Spectrum's Case Surveillance and Vital Registration tool

Severin Guy Mahiané*
Avenir Health, Glastonbury, CT, USA

Jeff Eaton
Imperial College, London, UK

Robert Glaubius
Avenir Health, Glastonbury, CT, USA

Kimberly Marsh
UNAIDS, Geneva, Switzerland

John Stover
Avenir Health, Glastonbury, CT, USA

March 11, 2019

Abstract

This document presents approaches to using Spectrum (AIM) model to estimate: a) the proportion of people living with HIV (PLHIV) who have been diagnosed, b) the mean CD4 at diagnosis, and c) the mean time from infection to diagnosis for these individuals.

1 A Simple Model for HIV infected individuals

In this paragraph, we consider a birth cohort in a population in which HIV infection is spreading over time. Let us assume that we are following the cohort of people alive between times 0 and t. Let w, v and s be the respective times of infection, diagnosis and treatment initiation; and let $S(t)$ be the susceptible population and let $I_{un}(t,w)$, $I_{du}(t,w)$, $I_{dt}(t,v,w)$ be the undiagnosed, diagnosed untreated, and diagnosed and treated infected populations, respectively. Figure 1 illustrates the flow of individuals in our population in the compartments considered here. That flow can be described by the following system:

$$\begin{aligned}
\frac{\partial S(t)}{\partial t} &= -\left(\lambda(t)+\mu(t)\right)S(t) \\
I_{un}(t,t) &= \lambda(t)S(t) \\
\frac{\partial I_{un}}{\partial t}+\frac{\partial I_{un}}{\partial w} &= -\left(m_{un}(t,w)+\delta(t,w)+\mu(t)\right)I_{un}(t,w) \\
I_{du}(t,t,w) &= \delta(t,w)I_{un}(t,w) \\
\frac{\partial I_{du}}{\partial t}+\frac{\partial I_{du}}{\partial w}+\frac{\partial I_{du}}{\partial v} &= -\left(m_{un}(t,w)+\eta(t,v,w)+\mu(t)\right)I_{du}(t,v,w) \\
I_{dt}(t,t,v,w) &= \eta(t,v,w)I_{du}(t,v,w) \\
\frac{\partial I_{dt}}{\partial t}+\frac{\partial I_{dt}}{\partial w}+\frac{\partial I_{dt}}{\partial v}+\frac{\partial I_{dt}}{\partial s} &= -\left(m_{dt}(t,v,w)+\mu(t)\right)I_{dt}(t,s,v,w)
\end{aligned} \tag{1}$$

where λ is the incidence function; μ is the background mortality; δ is the diagnosis rate; m_{un} is the mortality among HIV infected and undiagnosed; m_{du} is the mortality among infected, diagnosed and untreated; η is the treatment initiation among diagnosed individuals; and m_{dt} is the mortality rate among infected, diagnosed and treated individuals. This shows that, in order to tract infection, diagnosis and treatment initiation, a minimum of 10 compartment should be considered by age or risk group. In order to avoid this, we consider instead a simplified version in which infection, diagnosis and treatment initiation times are not tracked. The system reduces to a system of ODE:

$$\begin{aligned}
\frac{dS(t)}{dt} &= -\left(\lambda(t)+\mu(t)\right)S(t) \\
\frac{d\tilde{I}_{un}}{dt} &= \lambda(t)S(t)-\left(\tilde{m}_{un}(t)+\tilde{\delta}(t)+\mu(t)\right)\tilde{I}_{un}(t) \\
\frac{d\tilde{I}_{du}}{dt} &= \tilde{\delta}(t)\tilde{I}_{un}(t)-\left(\tilde{m}_{un}(t)+\tilde{\eta}(t)+\mu(t)\right)\tilde{I}_{du}(t) \\
\frac{d\tilde{I}_{dt}}{dt} &= \tilde{\eta}(t)\tilde{I}_{du}(t)-\left(\tilde{m}_{dt}(t)+\mu(t)\right)I_{dt}(t)
\end{aligned} \tag{2}$$

where the symbol ˜ put on top of population type, indicates that a sum was taken over all the possible infection time, diagnosis time and/or treatment initiation; and the the overall rates in the populations of interest. The solution to (2) can be otained from the solution of (1) if we have:

$$\tilde{\delta}(t)=\frac{\int_0^t \Lambda(w)M_0(t,w)\delta(t,w)dw}{\int_0^t \Lambda(w)M_0(t,w)dw} \tag{3}$$

$$\tilde{m}_{un}(t) = \frac{\int_0^t \Lambda(w) M_0(t,w) m_{un}(t,w) dw}{\int_0^t \Lambda(w) M_0(t,w) dw} \tag{4}$$

$$\tilde{m}_{dt}(t) = \frac{\int_0^t \Lambda(w) \int_w^t M_0(v,w)\delta(v,w) M_1(t,v,w)\eta(t,v,w) dv dw}{\int_0^t \Lambda(w) \int_w^t M_0(v,w)\delta(v,w) M_1(t,v,w) dv dw} \tag{5}$$

$$\tilde{\eta}(t) = \frac{\int_0^t \Lambda(w) \int_s^t \int_w^s M_0(v,w)\delta(v,w) M_1(s,v,w)\eta(s,v,w) M_2(t,s,w) m_{dt}(t,s,w) ds dv dw}{\int_0^t \Lambda(w) \int_s^t \int_w^s M_0(v,w)\delta(v,w) M_1(s,v,w)\eta(s,v,w) M_2(t,s,w) ds dv dw} \tag{6}$$

where $\Lambda(w) = e^{-\int_0^w \lambda(\zeta)d\zeta}\lambda(w)$, $M_0(v,w) = e^{-\int_w^v (m_{un}+\delta)(\zeta,w)d\zeta}$, $M_1(s,v,w) = e^{-\int_v^s (m_{un}(\zeta,w)+\eta(t,\zeta,w))d\zeta}$ and $M_2(s,v,w) = e^{-\int_v^s (m_{dt}(s,\zeta,w))d\zeta}$.

We can obtain the time to diagnosis and CD4 at diagnosis using formulae similar to (3) and (4). In fact, the mean time from infection to diagnosis is given by (7) and, if $g(t,w)$ gives the CD4 trajectory as a function of infection time, then the mean CD4 for newly diagniosed individuals, $\tilde{g}$, is given by (8).

$$\tilde{T}_d(t) = \frac{\int_0^t \Lambda(w) M_0(t,w)\delta(t,w)(t-w) dw}{\int_0^t \Lambda(w) M_0(t,w)\delta(t,w) dw} \tag{7}$$

$$\tilde{g}(t) = \frac{\int_0^t \Lambda(w) M_0(t,w)\delta(t,w) G(t,w) dw}{\int_0^t \Lambda(w) M_0(t,w)\delta(t,w) dw} \tag{8}$$

Now, we propose to approximate the solution to the System (3)-(6) using the following scheme:

$$\begin{aligned}
S_{t+\tau} &= S_t E_{0,t} && (9)\\
I_{un,t+\tau} &= I_{un,t} E_{1,t} + \frac{\lambda_t \tau S_t}{6}\left(E_{1,t} + 4E_0^{\frac{1}{2}} E_{1,t}^{\frac{1}{2}} + E_{0,t}\right) && (10)\\
I_{du,t+\tau} &= I_{du} E_{2,t} + \frac{\tilde{\delta}_t \tau I_{un,t}}{6}\left(E_{2,t} + 4E_2^{\frac{1}{2}} E_{1,t}^{\frac{1}{2}} + E_{1,t}\right)\\
&\quad + \frac{\lambda_t \tilde{\delta}_t \tau^2 S_t}{36}\left(E_{2,t} + 4E_2^{\frac{1}{2}} E_{1,t}^{\frac{1}{2}} + E_{1,t} + 2\left(E_{2,t}^{\frac{1}{2}} + 4E_{2,t}^{\frac{1}{4}} E_{1,t}^{\frac{1}{4}} + E_{1,t}^{\frac{1}{2}}\right)\right) && (11)\\
I_{dt,t+\tau} &= I_{dt} E_{3,t} + \frac{\tilde{\eta}_t \tau I_{du,t}}{6}\left(E_{3,t} + 4E_3^{\frac{1}{2}} E_{2,t}^{\frac{1}{2}} + E_{2,t}\right)\\
&\quad + \frac{\tilde{\delta}_t \tilde{\eta}_t \tau^2 I_{un,t}}{36}\left(E_{3,t} + 4E_3^{\frac{1}{2}} E_{2,t}^{\frac{1}{2}} + E_{2,t} + 2\left(E_{3,t}^{\frac{1}{2}} + 4E_{3,t}^{\frac{1}{4}} E_{2,t}^{\frac{1}{4}} + E_{2,t}^{\frac{1}{2}}\right)\right) && (12)
\end{aligned}$$

where $E_{0,t} = e^{\lambda_t \tau}$, $E_{1,t} = e^{-\left(\tilde{m}_{un,t}+\tilde{\delta}_t\right)\tau}$, $E_{2,t} = e^{-(\tilde{m}_{du,t}+\tilde{\eta}_t)\tau}$ and $E_{3,t} = e^{-\tilde{m}_{dt,t}\tau}$, and the subscripts indexed by time (t or $t+\tau$) indicate the dependency with respect to time.

Note that the background mortality was dropped from (9)-to-(12) because we are only interested in proportions; the main simulation is performed using Spectrum's AIM. In fact, for each bith cohort, we solve the system (9)-to-(12) with the initial condition $S_0 = 1$, $I_{un,0} = I_{du,0} = I_{dt,0} = 0$ then, for each time, the proportion of undiagnosed, q_t is given by (13).

$$q_t = \frac{I_{un,t}}{I_{un,t} + I_{du,t} + I_{dt,t}}, \tag{13}$$

and the proportion of PLHIV newly diagnosed (between t and $t+\tau$), $p_{new,t+\tau}$ is given by (14).

$$p_{new,t+\tau} = \frac{I_{du,t+\tau} + I_{dt,t+\tau} - I_{du,t}E_{2,t} - I_{dt,t}E_{3,t}}{I_{un,t+\tau} + I_{du,t+\tau} + I_{dt,t+\tau}}, \tag{14}$$

In this work, we assume that the diagnosis rate is proportional to mortality rate in absence of treatment, i.e.

$$\delta(v,w) = \bar{\delta}(v) m_{un}(v,w), \quad \forall w, v: \; v \geq w,$$

where

$$\bar{\delta}(v) = \Gamma(v - t_0, z_1, 1)$$

and Γ is a Gamma cummulative distribution function with shape z_1 and scale 1, z_2 is a scale factor, and t_0 is the year when the first diagnosis was observed.

Formulae (3)-to-(8) do not appear very practical because they involve integrals of functions. Their discretized versions are used instead. Furthermore, the closed forms of functions $\delta(t,w)$, $g(t,w)$, $m_{un}(t,w)$, $\eta(t,v,w)$ and $m_{dt}(t,v,w)$ are not directly available from Spectrum. We discuss their construction in Section 1.1.

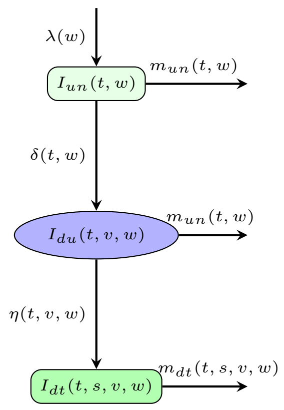

Figure 1: Flow chart for the HIV infected population. $\lambda(w)$ is the incidence hazard rate at time w, $I_{un}(t,w)$ is the infected population not yet diagnosed, $I_{du}(t,w)$ is the infected diagnosed but who have not started treatment yet, $I_{dt}(t,w)$ is the infected population on treatment, $\delta(t,w)$ is the rate at which individuals are diagnosed, $m_{un}(t,w)$ is the mortality rate for undiagnosed, $m_{dt}(t,s,v,w)$ is the mortality for those who started treatment at time s and $\eta(t,v,w)$ is the treatment initiation rate.

1.1 CD4 trajectories, mortality and diagnosis rates

We obtain the $CD4$ trajectory as a function of age at infection using Spectrum progression rates as follows. Let a_0 be the age at infection and c_0 the CD4 count at infection.

Define the age category, $a1$, the CD4 category, $c1$, the time of change of CD4 category, u, the time of change of category, v. Now, for each CD4 category $c1$, let C_{c1} be the largest CD4 value for that category and let $\nu_{a1,c1}$ be the CD4 progression rate from CD4 category $c1$ to the lower CD4 category, $c1+1$, for individuals in the age category $a1$. Let A_1 denote the maximum age for individuals in the age category $a1$.

Set $a = a_0$, $c = c_0$ and repeat the following steps.

Algorithm 1

Step 1 Calculate:

- Time to CD4 category change, $u = -\frac{1}{\nu_{a1,c1}} \log\left(\frac{C_{c1+1}}{C_{c1}}\right)$
- Time to age category change, $v = A_{a1+1} - a$

Step 2 Do:

- If progression to the next CD4 category occurs before age category change, i.e. $u < v$, then:

$$c1 = c1 + 1, \quad a = a + u, \quad t = t + u$$

- Else (change of age caterogy happens before CD4 category threshold), then:

$$a = A_{a1+1}, \quad , a1 = a1 + 1, \quad t = t + v$$

Step 3 Do:

- If minimum CD4 or maximum age is reached, then Stop.
- Else go to Step 1.

The above algorithm can give the CD4 trajectory of infected people as a function of both time (or, equivalently, age) and CD4 at infection, $\tilde{f}.(t,w,c_0)$, where $(\cdot = x$ for men or y for women). In order to obtain the trajectory as a function of time only, we integrate that function over the CD4 distribution at infection, using Spectrum assumption regarding that distribution; i.e.

$$\tilde{f}.(t,w) = \sum_{c_0=1}^{c_{max}} \tilde{f}.(t,a,w,c_0)\pi_{a-t+w,c_0},$$

where π_{a-t+w,c_0} is the probability that an individual who became infected at age $a-t+w$ had CD4 count in the category c_0 at infection. Figures 2 (a) and (b) displays CD4 trajectories as a function of age at infection for both women and men, obtained as described in Algorithm 1 for developed countries, using Spectrums assumptions.

In order to obtain mortality rates as a function of time and age at infection for undiagnosed individuals, one can follow the the CD4 trajectory and assign the mortality rate that corresponds to the CD4 category. More precisely, to get mortality for individuals infected at age $a0$ (or time w), we first obtain their CD4 at time t, $\tilde{f}.(t,w)$ then, we use the $CD4$ category obtain that way to get the mortality as assumed by Spectrum. Figures 3 (a) and (b) illustrates mortality rates as a function of age at infection for women and men, respectively. Similarly, we can obtain mortality as a function of age at infection and age at ART initiation; Figures 4 (a)and (b) illustrate the change in mortality rate as a function of age at ART initiation, for women in developed countries who became infected at 15 and 30 years, respectively.

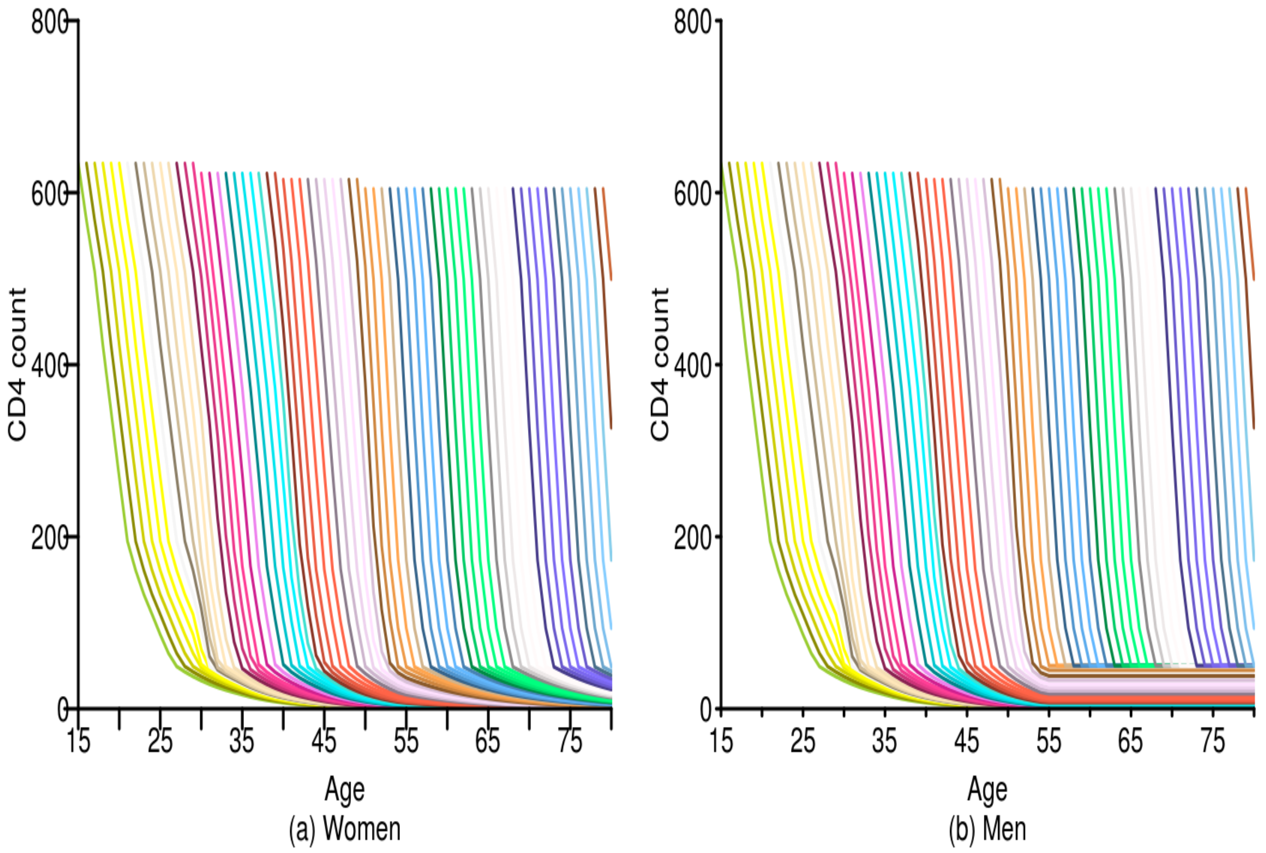

Figure 2: CD4 decline as a function of age at infection for women (a) and men (b).

Finally, because we can track $CD4$ trajectories as a function of time since infection, one can easily specify the treatment initialtion rate $\eta(t, v, w)$ as a function of WHO guidelines. More specifically, we assume that, for diagnosed individuals, the time to ART initiation when eligibility criteria are met follow an Exponential distribution with mean 3 months. Moreover, diagnosed individuals who do not meet treatment eligibility also intiate treatment at a rate proportional to HIV related mortality; i.e., if μ_0 is HIV related mortality at treatment initiation and μ_c is mortality rate of noneligible individuals in the CD4 category c, then treatment itiation rate for these individuals is $3\frac{\mu_c}{\mu_0}\frac{e^{\epsilon}}{1+e^{\epsilon}}$.

1.2 Incidence Options

Double and single logistic curves. Prior this work, the principle supporting the CSVAR fitting tool was to choose a family of parametric curves, vary the parameter values and retain the set that provides the best fit to the data, using the either maximum likelihood estimation method or by minimizing the Chi-squared distance (see [2]). The family of candidate curves consisted of double logistic and single logistic functions (see [2]). The double logistic curve was parameterized by 5 parameters while the single logistic was parameterized by two parameters. To be more specific, the double logistic incidence curve was given by (15)

$$\lambda(t) = \frac{e^{\alpha(t-t_0)}}{1+e^{\alpha(t-t_0)}}\left(2a\frac{e^{-\beta(t-t_0)}}{1+e^{-\beta(t-t_0)}}+b\right), \quad t_0, a, b, \alpha, \beta > 0 \tag{15}$$

and the single logistic incidence curve was given by (16)

$$\lambda(t) = \frac{e^{-c+\alpha(t-1970)}}{1+e^{-c+\alpha(t-1970)}}, \quad a, b, \alpha, \beta > 0 \tag{16}$$

This contains functions flexible enough to capture most HIV epidemic trends. However, for a handful of countries, the best curve obtained from that family was not satisfactory.

In the Bayesian framework, the double logistic model is fitted with the following prior distribution on its parameters:

$$\begin{gathered}\log(\alpha) \sim \mathcal{N}(-2,2), \quad \log(\beta) \sim \mathcal{N}(-2,2), \quad \log(t_0-1970) \sim \mathcal{N}(\log(10),1)\\ \log(-\log(a)) \sim \mathcal{N}(2.44,2), \quad \log(-\log(b)) \sim \mathcal{N}(1.95,2)\end{gathered} \tag{17}$$

while the single logistic model is fitted with the prior distributions on its parameters:

$$\log(c) \sim \mathcal{N}(3,1), \quad \log(\alpha) \sim \mathcal{N}(-5,5) \tag{18}$$

Second Order Segmented polynomials. We included second order segmented polynomial functions (see [3]) as an option, to circumvent the limitations of the single and double logistic curves. With this choice, we can estimate the position of knots.

Although this family of functions is very flexible, the number of parameters needed can be relatively large. Furthermore, the functions in that family are not naturally constrained to be non negative. One can

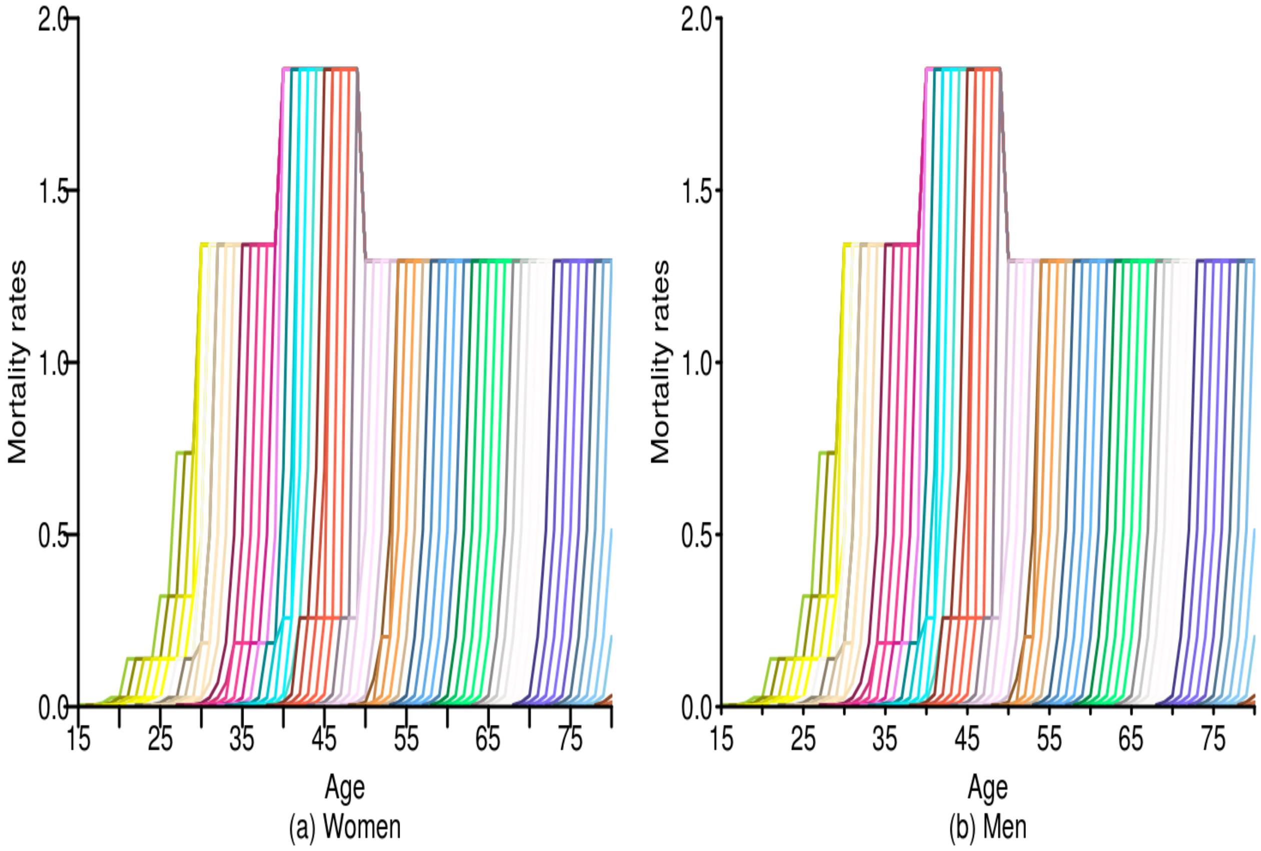

Figure 3: Mortality rates as a function of age at infection for infected an untreated women (a) and men (b).

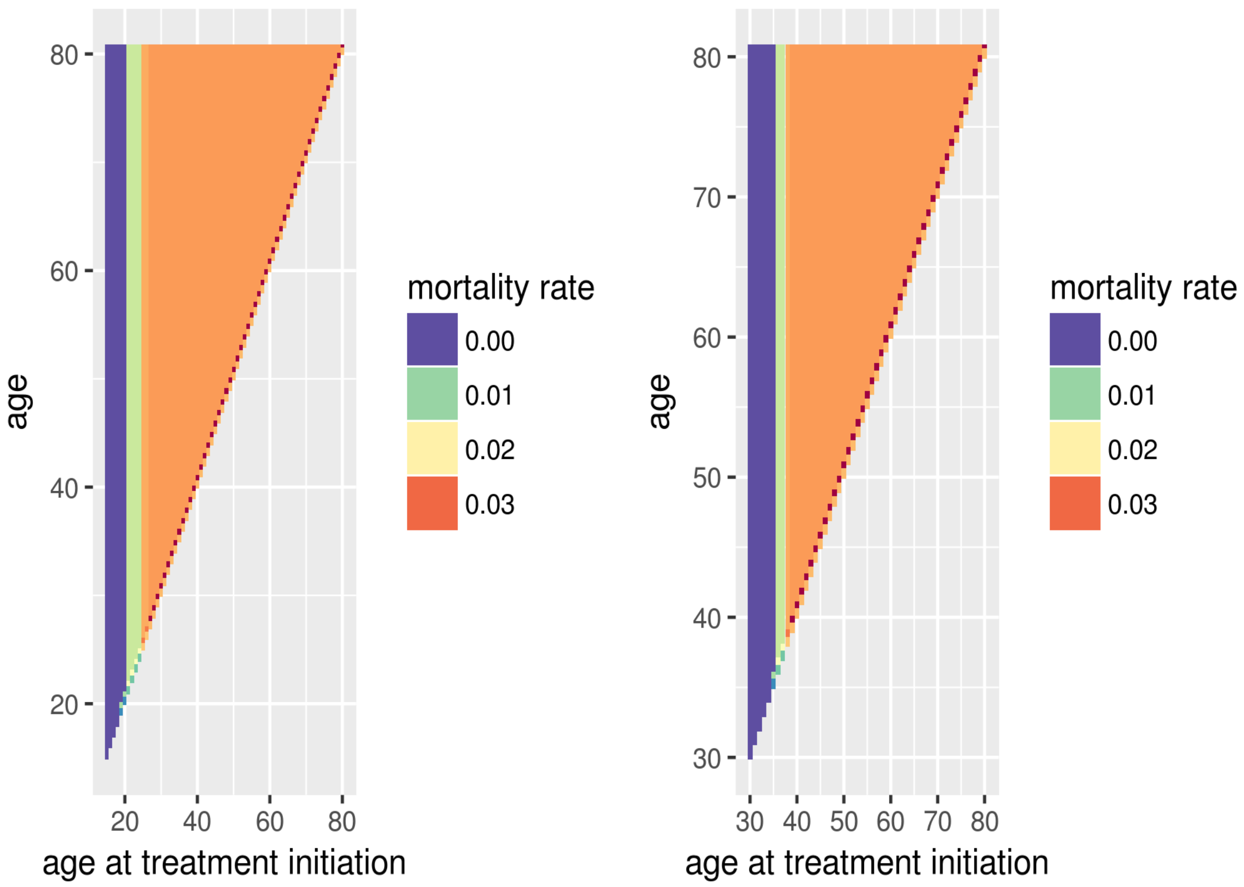

Figure 4: Mortality rates as a function of age at ART initiation for women in developed countries aged 15 (a) and 30 (b) when they became infected.

use the augmented Lagragian to enforce constraints on the incidence or, alternatively, in order not increase the complexity of the problem, we propose to use a transformation of the spline. In fact, we used the transformtation $x \mapsto i_{max}\frac{x^2}{1+x^2}$, where i_{max} is a prior bound on the incidence rate; i.e., for this model, the incidence is given by (19):

$$\lambda(t) = \lambda_{max}\frac{i^2(t)}{1+i^2(t)} \tag{19}$$

were λ_{max} is the largest possible value allowed for the incidence rate, and $i(t) = a_k + b_k(t-t_k) + c_k(t-t_k)^2$, for $t \in (t_{k-1}, t_k)$ t_0 is the start year of the epidemic, $a_k, b_k, k = 0, 1, 2, 3$ and $t_k, k = 1, 2$ are parameters to be estimated. In Bayesian analyses this model was fitted with the following prior distributions:

$$\begin{gathered} a_0 \sim \mathcal{N}(-30, 2), \quad b_0 \sim \mathcal{N}(-10, 2), \quad c_k \sim (-1)^{(k+1)}\mathcal{N}(0.005, 1) \\ \zeta_k\left(\frac{t_k - t_{k-1}}{t_{max} - t_0}\right) \sim \mathcal{N}(0, \tfrac{1}{3}), \quad k = 1, 2, 3 \end{gathered} \tag{20}$$

where $\zeta = (\zeta_1, \zeta_2, \zeta_3)$ is the inverse of the transformation $(x_1, x_2, x_3) \mapsto \left(\frac{e^{x_1}}{1+\sum_{k=1}^{3} e^{x_i}}, \frac{e^{x_2}}{1+\sum_{k=1}^{3} e^{x_i}}, \frac{e^{x_3}}{1+\sum_{k=1}^{3} e^{x_i}}\right)$.

Transmission model using the *rlogistic* function. Instead of directly modelling the HIV incidence rate directly, we also consider modelling the transmission rate $r(t)$, as in the Estimation Projection Package (EPP) model [1]. In this case, the incidence rate is given by (21).

$$\lambda(t) = r(t)p(t)\left(1 - 0.7\kappa(t)\right) \tag{21}$$

where $p(t)$ and $\kappa(t)$ are the prevalence and ART coverage at time t, respectively, and 0.7 is the average reduction in transmission per additional person on ART. We use a logistic function to model the logarithm of $r(t)$, termed *rlogistic* with four parameters:

$$\log r(t) = r_0 - (r_\infty - r_0)\frac{1}{1+\exp\left(-\alpha(t - t_{mid})\right)}$$

where $\exp(r_0)$ is the initial exponential growth rate of the epidemic, $\exp(r_\infty)$ is the equilibrium value for $r(t)$, α is the rate of change of $r(t)$ in the log-scale, and t_{mid} is the inflection point. For this model, we additionally specify a fifth parameter, ι, as the incidence rate at time $t = t_0$, providing the initial pulse of infections. This model is fitted with prior distributions on its parameter:

$$\begin{gathered} r_0 \sim \mathcal{N}\left(\log(0.5), 0.5\right), \quad r_\infty \sim \mathcal{N}\left(\log(0.09), 0.3\right) \quad \log(\alpha) \sim \mathcal{N}\left(\log(0.2), 0.5\right), \\ t_{mid} \sim \mathcal{N}\left(1993, 5\right), \quad \iota \sim \mathcal{N}\left(-13, 5\right). \end{gathered} \tag{22}$$

2 Fitting procedures

2.1 Previous version

In the previous approach, it was assumed that data consist of numbers of new diagnoses $(n_i(t_{ij}),\ j = 1 \dots j_i)$, deaths $(n_d(t_{dj}),\ j = 1 \dots j_d)$ and PLHIV $(n_h(t_{hj}),\ j = 1 \dots j_h)$, where t_j, $(j \in \{j = 1 \dots j_i\} \cup \{j = 1 \dots j_d\} \cup \{j = 1 \dots j_h\})$ are the observation times. For the likelihood estimation approach, it was assumed that the observed numbers followed Poisson distributions and, under that assumption, maximizing the likelihood was equivalent to minimizing the loss function given by (23).

$$nlik(\theta) = -\sum_{u=i,d,h}\sum_{j=1}^{j_u}\left(n_u(t_{uj})\log(\widehat{n}_u(\theta, t_{uj})) - \widehat{n}_u(\theta, t_{uj})\right), \tag{23}$$

where we adopted the convention that the sum over an empty set is zero; θ is the vector of parameter values, and the hat over quantities indicates Spectrum predictions when the incidence curve is defined by θ.

For the minimum Chi-Squared distance method, the loss function was rather given by (24).

$$ks(\theta) = \sum_{u=i,d,h}\sum_{j=1}^{j_u}\frac{\left(n_u(t_{uj}) - \widehat{n}_u(\theta, t_{uj})\right)^2}{n_u(t_{uj})}, \tag{24}$$

Note that, in the previous version, it was not possible to get the number of new diagnoses directly from Spectrum. The number of new diagnoses was used to obtain a crude approximation of the number of new incident cases and vice versa.

2.2 New version

The models presented in Sections 1 allow avoiding the assumptions needed to derive the number of incident cases at the cost of adding two more parameters used to model the diagnosis rate as a function of time. We assume that the observed CD4 at diagnosis follow a Gamma distribution with parameter (α, β). Let $\theta = (\theta', \alpha, \beta, \epsilon)$ be the parameter under the new version (where θ' is the incidence parameter, and ϵ models treatment initiation for in dividuals who don't meet WHO eligibility criteria). Now, we assume that mean CD4 can only be measured in years when new diagnoses are observed and their number in greater that 1.

Let $n_a(t_{aj})$, $j = 1 \dots j_a$ be the number of people on *ART* reported by the country. We assume here that the numbers observed follow Poisson distributions, and that the mean CD4 at diagnosis follow Gamma distributions. If we keep the notations of the previous paragraphs, the loss function given by (23) becomes:

$$
\begin{aligned}
nlik(\theta) \quad = \quad & -\sum_{u=i,d,h}\sum_{j=1}^{j_u}\Big(n_u(t_{uj})\log(\widehat{n}_u^(\theta,t_{uj})) - \widehat{n}_u(\theta,t_{uj})\Big) \\
& -\sum_{j=1}^{j_a}(n_a(t_{aj})\log(\widehat{n}_a(\theta,t_{aj})) - \widehat{n}_a(\theta,t_{aj})) \\
& -\sum_{j=1}^{j_d}(\alpha n_d(t_j)-1)\, g_j(t_j)\log(\widehat{g}_j(t_j)) - \alpha n_d(t_j)\widehat{g}_j(\theta,t_j),
\end{aligned} \tag{25}
$$

We fitted the models by maximizing the posterior likelihood, which is equivalent to minimizing:

$$
L(\theta) = -P_1(\theta') - P_2(z_1, z_2, \epsilon) + nlik(\theta), \tag{26}
$$

where P_1 is determined by (17), (18), (20) or (22), and P_2 is such that $\log(z_1) \sim \mathcal{N}(2.7, 10)$, $\log(z_2) \sim \mathcal{N}(1.5, 10)$, and $\epsilon \sim \mathcal{N}(0, 1)$.

Kernel Hamiltonian Monte Carlo [4] was implemented as an option for the calibration.

References

1. Le Bao. A new infectious disease model for estimating and projecting hiv/aids epidemics. *Sexually transmitted infections*, 88 Suppl 2:i58–i64, December 2012.

2. Severin G Mahiane, Kimberly Marsh, Kelsey Grantham, Shawna Crichlow, Karen Caceres, and John Stover. Improvements in spectrum's fit to program data tool. *AIDS*, 31:S23–S30, 2017.

3. Severin Guy Mahiané and Oliver Laeyendecker. Segmented polynomials for incidence rate estimation from prevalence data. *Stat Med*, 36:334–344, January 2017.

4. Heiko Strathmann, Dino Sejdinovic, Samuel Livingstone, Zoltan Szabo, and Arthur Gretton. Gradient-free hamiltonian monte carlo with efficient kernel exponential families.
